# Supplementary material for: Biochemical engineering of 5hmdC-DNA using a Tet3 double-mutant
Source: Comput Struct Biotechnol J. 2025 Dec 28;31:389–96. doi: 10.1016/j.csbj.2025.12.021 (PMC12830177; doi:10.1016/j.csbj.2025.12.021)
Supplement: Supplementary file 1 — Supplementary material [file mmc1.pdf]

Supplemental Information 1 – Additional Results

Biochemical engineering of 5hmdC-DNA using a Tet3 double mutant

Hanife Sahin, Shariful Islam, A Hyeon Lee, Irene Ponzo, Kilian Winiger, Andreas Reichl, Thomas Carell, Pascal Giehr

Additional results

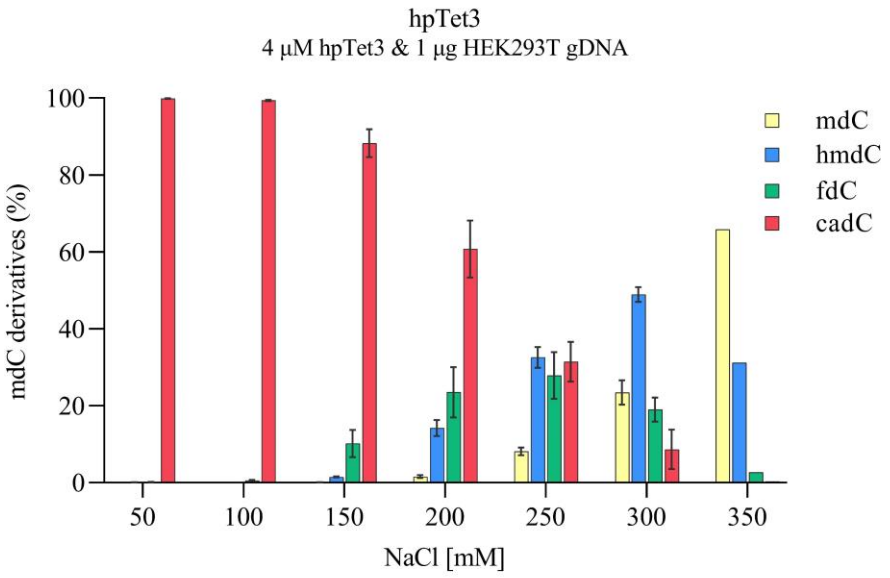

Figure S1. Effect of different NaCl concentrations on the catalytic activity of hpTet3. For each condition, 1 µg of HEK293T genomic DNA was incubated with 4 µM recombinant hpTet3 protein with the respective NaCl concentration at 37°C for 1 h. Samples were analyzed by UHPLC-QQQ-MS/MS. Values represent mean ± SD, n =3 for NaCl concentrations ranging from 50 mM–300 mM, n=1 for NaCl concentration of 350 mM.

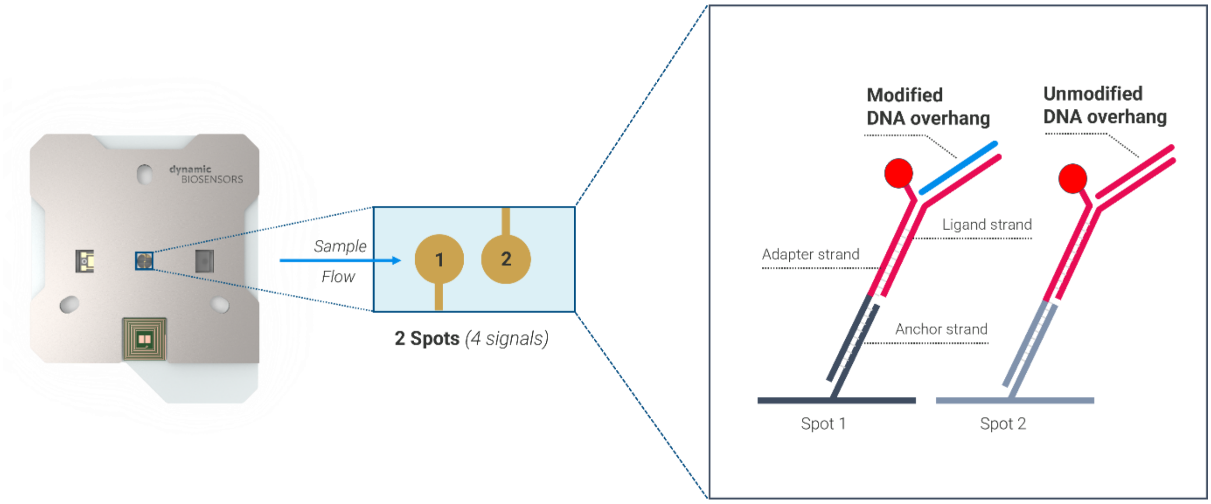

Figure S2. heliX Adapter chip setup. Binding kinetics are measured on two gold spots within the microfluidic channel of a heliX® chip. Graphical representation of Spot 1 used as measurement spot, in which the dsDNA overhang contains 5mdC modification, whereas Spot 2 is used as reference spot, with unmodified dsDNA overhang.

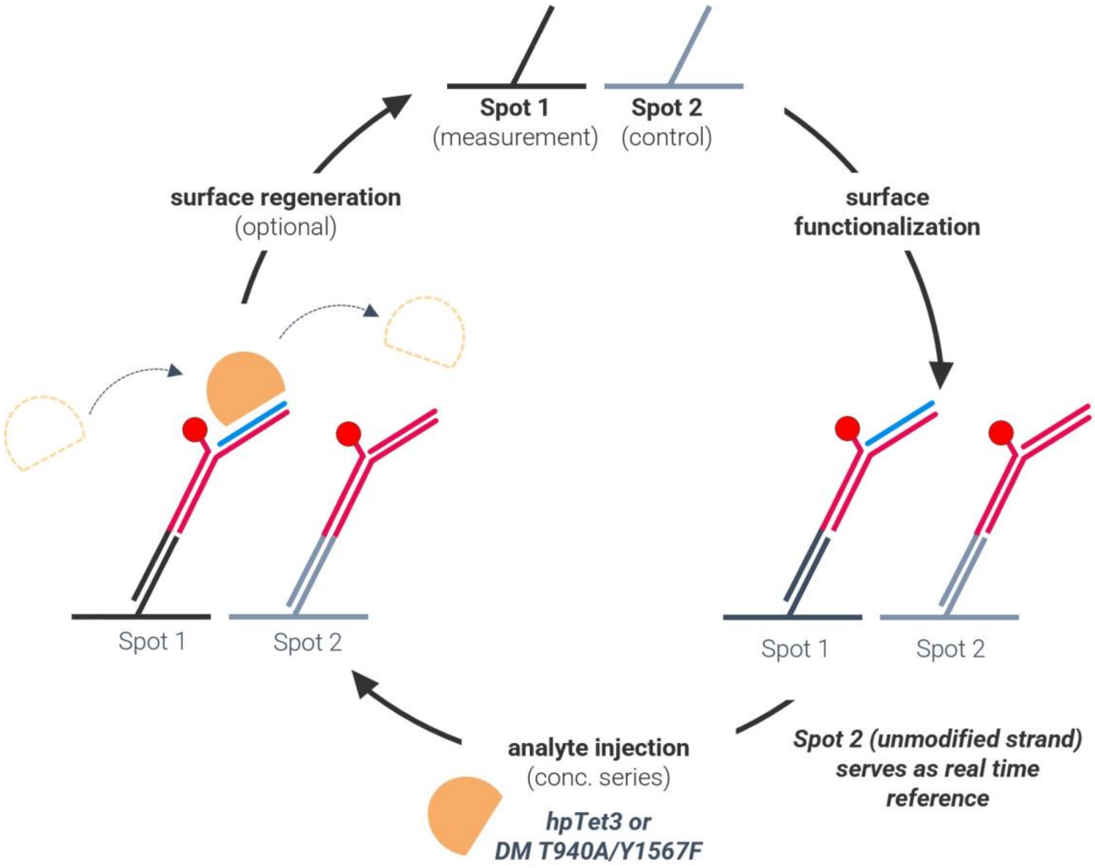

Figure S3. Representation of the switchSENSE® experimental workflow, consisting of 3 steps: (1) immobilization of the DNA ligands on the biochip surface; (2) Protein binding: association and dissociation kinetics are measured; (3) surface regeneration: injection of a high pH solution allows to remove the DNA ligands from the surface, and the biochip is ready to be functionalized again with fresh DNA ligand strands.

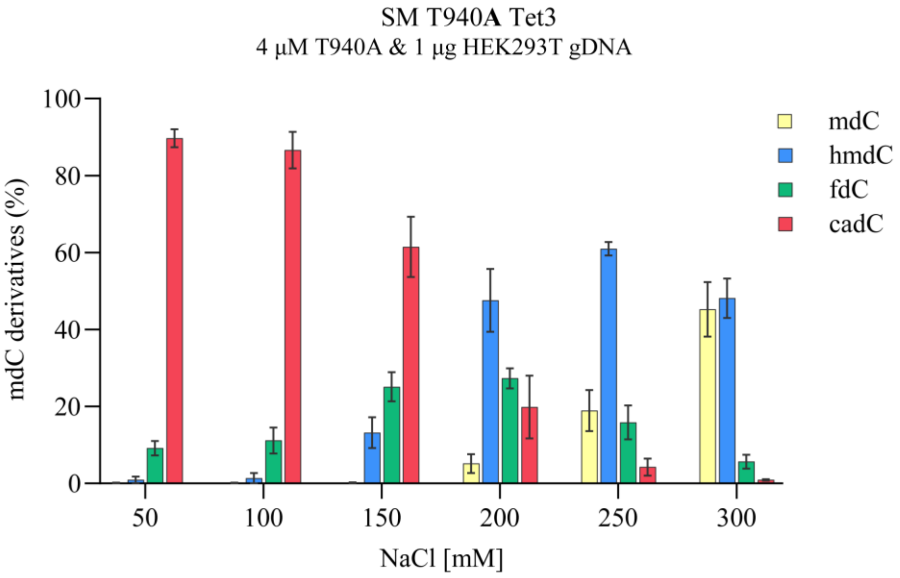

Figure S4. Impact of different NaCl concentrations on the catalytic activity of SM T940A Tet3. 1 µg of HEK293T genomic DNA was incubated with 4 µM recombinant SM T940A Tet3 protein with different NaCl concentrations at 37°C for 1 h. Samples were analyzed by UHPLC-QQQ-MS/MS. Values represent mean ± SD, n =3.

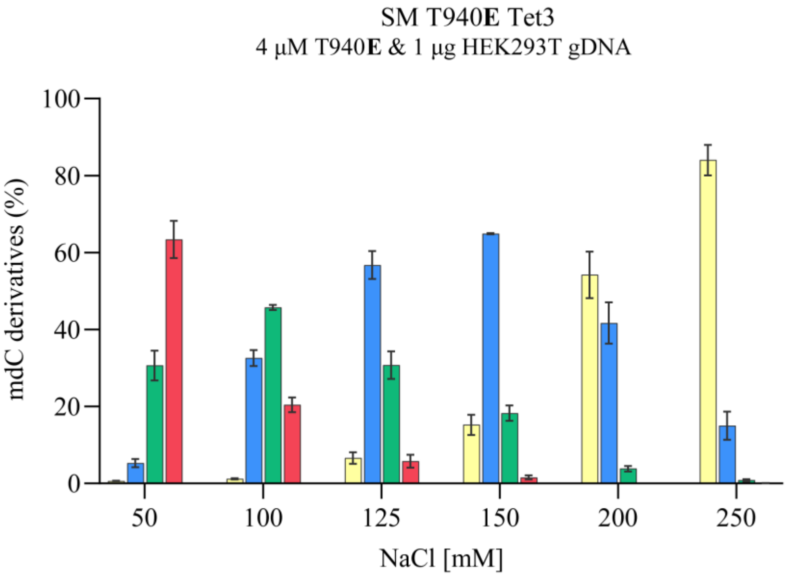

Figure S5. Impact of different NaCl concentrations on the catalytic activity of SM T940E Tet3. 1 µg of HEK293T genomic DNA was incubated with 4 µM recombinant SM T940E Tet3 protein with different NaCl concentrations at 37°C for 1 h. Samples were analyzed by UHPLC-QQQ-MS/MS. Values represent mean ± SD, n =4.

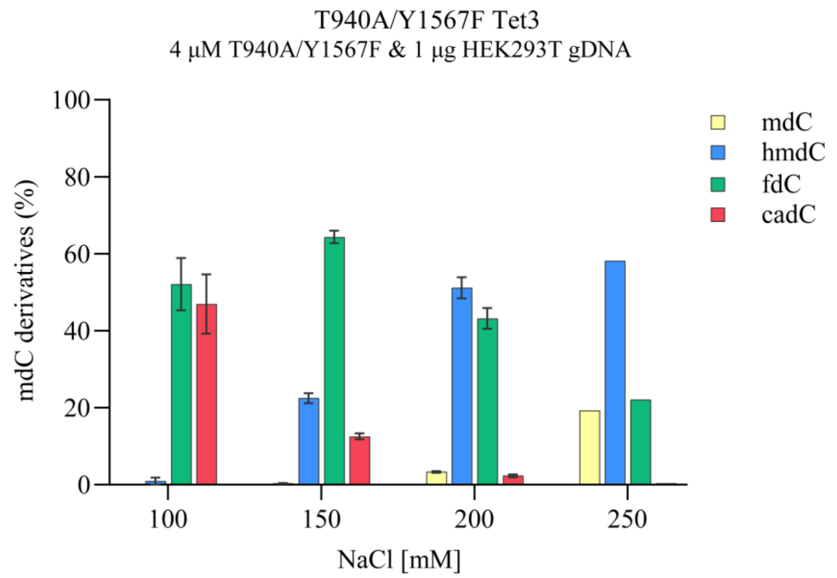

Figure S6. Impact of different NaCl concentrations on the catalytic activity of DM T940A/Y1567F Tet3. 1 µg of HEK293T genomic DNA was incubated with 4 µM recombinant DM T940A/Y1567F Tet3 protein with different NaCl concentration at 37°C for 1 h. Samples were analyzed by UHPLC-QQQ-MS/MS. Values represent mean ± SD, n =2 for NaCl concentrations of 100 mM, 150 mM, and 200 mM, n=1 for NaCl concentration of 250 mM.

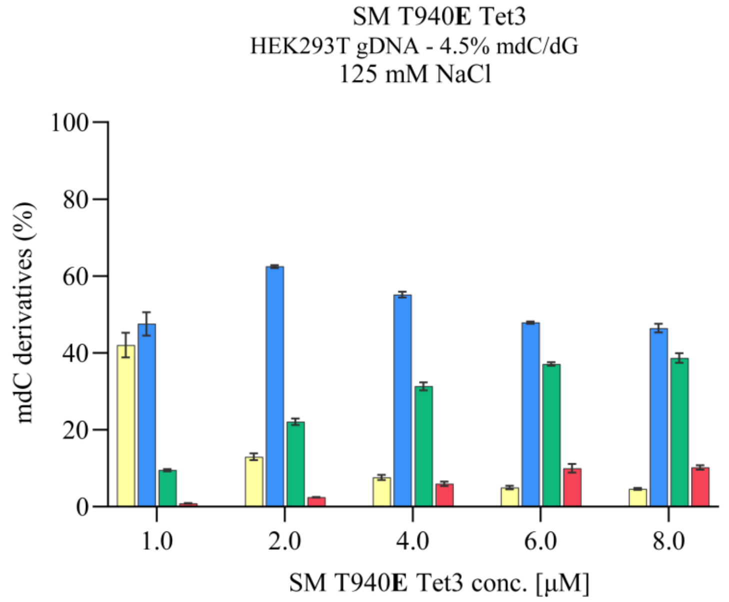

Figure S7. Impact of varying SM T940E Tet3 concentrations on the oxidation of 5mdC to 5hmdC, 5fdC, and 5cadC at 125 mM NaCl. For each reaction, 1 µg of human gDNA isolated from HEK293T cells was incubated with the respective amount of recombinant SM T1940E **Tet3** protein at 37°C for 1 h. Samples were analyzed by UHPLC-QQQ-MS. Values represent mean ± SD, n =4.

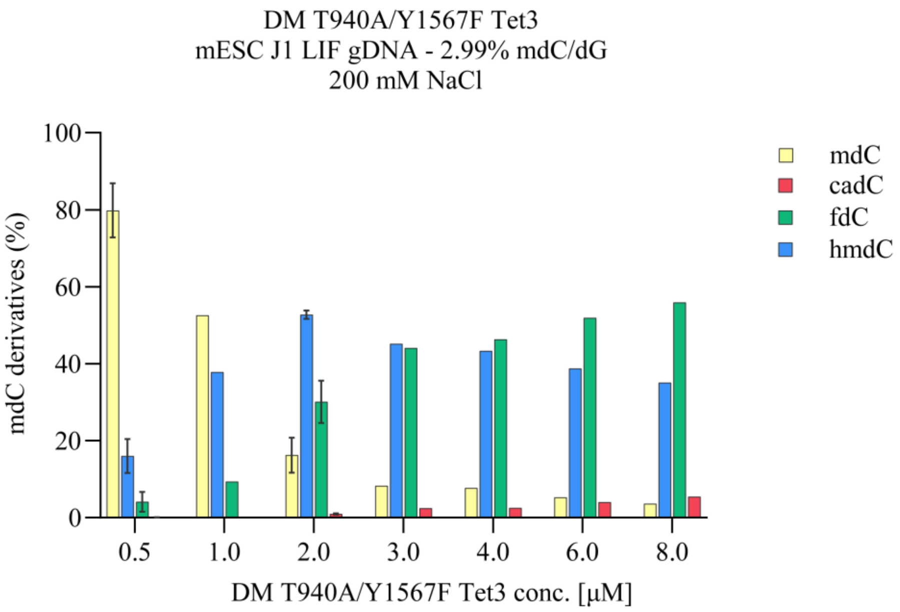

Figure S8. Impact of various DM T940A/Y1567F Tet3 concentrations on the oxidation of 5mdC to 5hmdC, 5fdC, and 5cadC at 200 mM NaCl. For each reaction, 1 µg of mouse gDNA isolated from J1 mouse embryonic stem cells cultivated in the primed state (mESC J1 LIF, primed for 3 days) was incubated with the respective amount of recombinant DM T940A/Y1567F Tet3 protein at 37°C for 1 h. Samples were analyzed by UHPLC-QQQ-MS. Values represent mean ± SD, n =2 for 0.5 µM and 2 µM DM T940A/Y1567F Tet3, n=1 for the remaining DM T940A/Y1567F Tet3 concentrations.

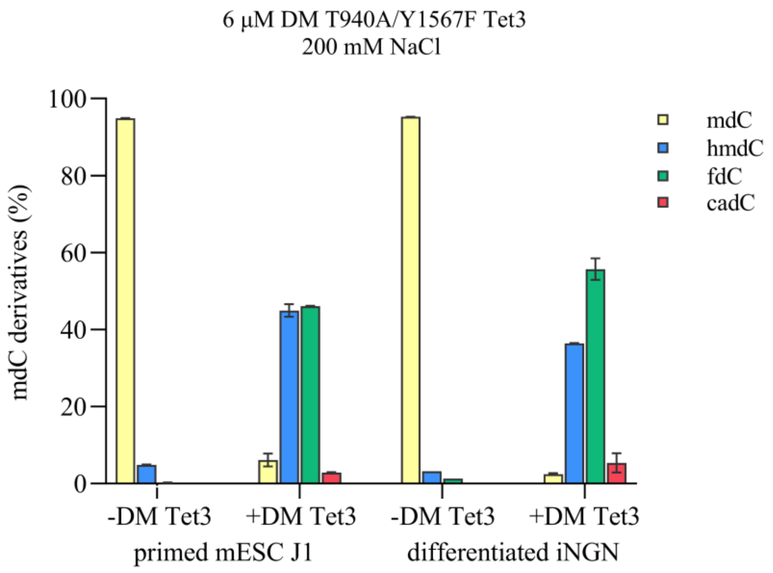

Figure S9. UHPLC-QQQ-MS-based absolute quantification of the catalytic activity of DM T940A/Y1567F Tet3 on genomic DNA containing naturally high 5hmdC levels. 1 µg of genomic DNA isolated from iNGNs differentiated for four days post-stimulation and mouse embryonic stem cells cultivated in the primed state for 7 days (mESC J1 LIF, 7 days) was incubated with 6 µM recombinant DM T940A/Y1567F Tet3 protein with 200 mM NaCl at 37°C for 1 h. Values represent mean ± SD, n =2.

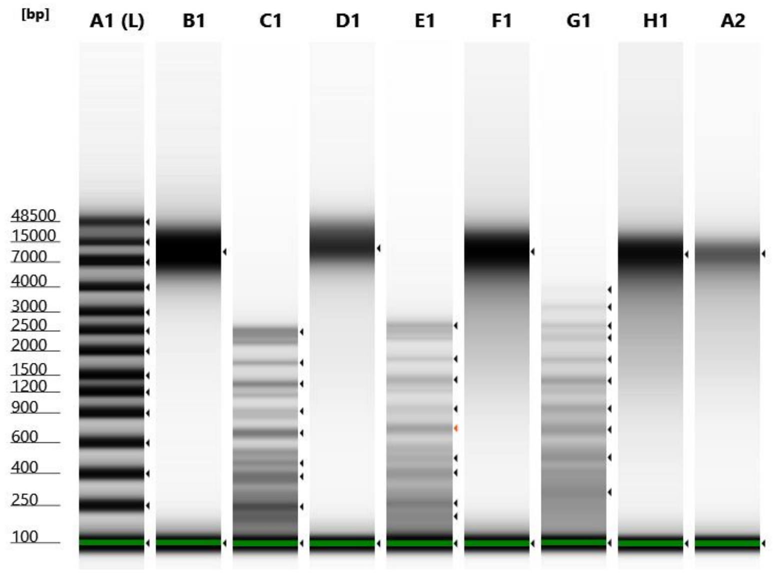

Figure S10. TapeStation analysis of MspI-digested lambda DNA substrates. Lambda DNA substrates, unmodified (LMD DNA), methylated (mdC-LMD), hydroxymethylated (hmdC-LMD), and glucosylated hydroxymethylated (glc-hmdC-LMD), were digested with the restriction enzyme MspI and analyzed using the Agilent 4150 TapeStation system. Digested samples (lanes C1 = dCpG, E1 = mdCpG, G1 = hmdCpG, A2 =glc-hmdCpG) were compared to their respective undigested controls (B1 = dCpG, D1 = mdCpG, F1 = hmdCpG, H1 = glc-hmdCpG). MspI preferentially cleaves at C/CGG sites. TapeStation analysis revealed that MspI efficiently fragmented unmodified, methylated, and hydroxymethylated lambda DNA, whereas glucosylated hydroxymethylated DNA was protected from cleavage due to glucose addition to hmdC, which blocks MspI recognition and cutting. A1 (L) = ladder i.e. size standard.

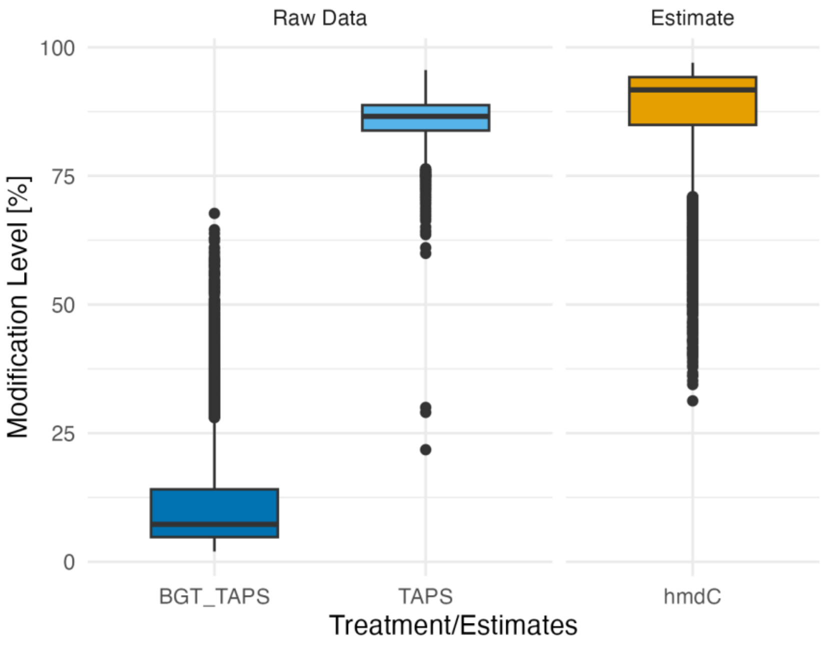

Figure S11. Tet-assisted pyridine borane sequencing (TAPS) and T4 beta glucosyl transferase TAPS (BGT-TAPS) sequencing results. Displayed are the modification distribution modified lambda LMD i.e. methylated, oxidized with DM-Tet3 and sodium borohydride reduction, after TAPS or BGT-TAPS, as well as the estimated hmdC level derived from the comparison of TAPS and BGT-TAPS.

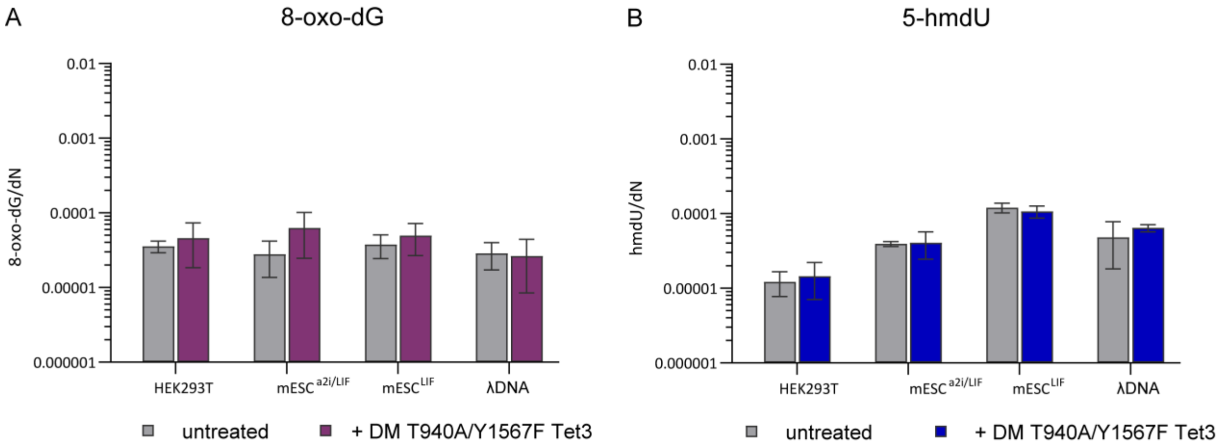

Figure S12. 8-oxo-dG (A) and hmdU (B) levels per nucleosides (dN) of genomic DNA before and after treatment with DM T940A/Y1567F Tet3 as quantified by UHPLC-QQQ-MS. Values represent mean ± SD, n =2.

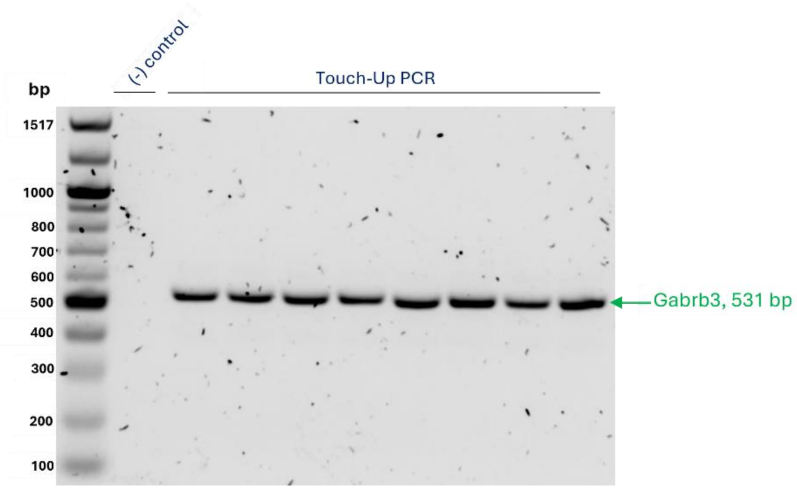

Figure S13. Amplifications of the PCR product, Gabrb3, by Touch-Up PCR. The PCR products were analysed on 1.2% agarose gels and compared with the 100 bp DNA Ladder (N3231L, New England Biolabs).

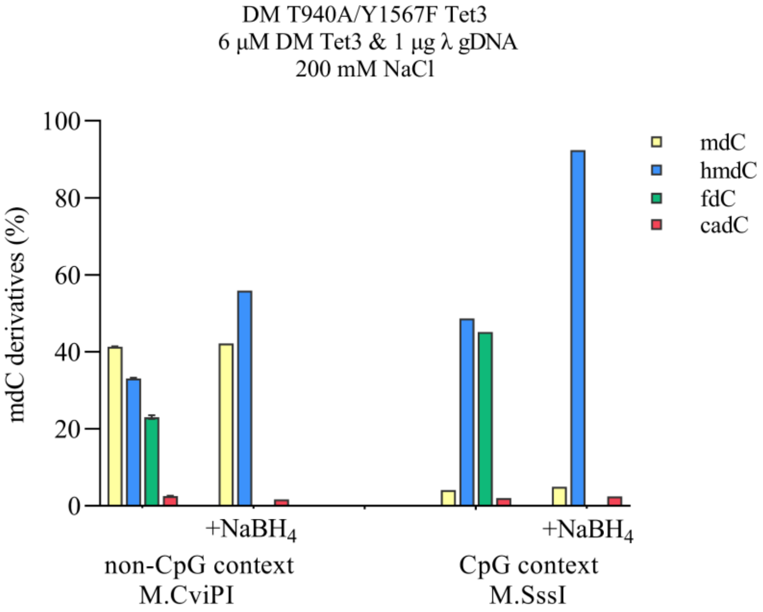

Figure S14. UHPLC-QQQ-MS-based absolute quantification of the catalytic activity of DM T940A/Y1567F Tet3 on lambda DNA methylated using methyltransferases in non-CpG context (M.CviPI) and CpG context (M.SssI) with subsequent sodium borohydride reduction. 1 μg of lambda DNA methylated with the respective methyltransferase was incubated with 6 μM recombinant DM T940A/Y1567F Tet3 protein with 200 mM NaCl at 37°C for 1 h. Samples were analyzed by UHPLC-QQQ-MS. Values represent mean ± SD, n =2 for DM Tet3 oxidation of M.CviPI methylated lambda DNA, n=1 for the remaining samples.

Table S1. Kinetic Rates Overview.

**hpTet3**	k_{on1} (E+6 $M^{-1}s^{-1}$)	k_{on2} (E+6 $M^{-1}s^{-1}$)	k_{off1} (E-3 s^{-1})	k_{off2} (E-3 s^{-1})	Rel. Amplitude k_{off1}, k_{off2} (%)	K_{d1} (nM)	K_{d2} (nM)
HE140	4.02 ± 2.55	0.12 ± 0.01	121 ± 6	0.45 ± 0.03	50, 50	30.2 ± 19.2	3.89 ± 0.23
HE200	0.32 ± 0.21	**0.02 ± 0.01**	159 ± 14	**0.09 ± 0.04**	35, **65**	492 ± 323	**4.00 ± 1.79**
HE300				N/D			
**DM**							
HE140	0.37 ± 0.09	0.37 ± 0.01	203 ± 13	0.84 ± 0.04	48, 52	552 ± 60.5	2.27 ± 0.12
HE200	2.04 ± 1.57	**0.08 ± 0.02**	81 ± 16	**0.74 ± 0.07**	14, **85**	39.8 ± 31.6	**9.42 ± 2.38**
HE300				N/D			
